# Enhancement of phenolics content and biological activities of longan (*Dimocarpus longan* Lour.) treated with thermal and ageing process

**DOI:** 10.1038/s41598-021-95605-3

**Published:** 2021-08-05

**Authors:** Preaploy Hong-in, Waranya Neimkhum, Chanun Punyoyai, Suwannee Sriyab, Wantida Chaiyana

**Affiliations:** 1 Master’s Degree Program in Cosmetic Science, Faculty of Pharmacy, Chiang Mai University, Chiang Mai, 50200 Thailand; 2 Department of Pharmaceutical Sciences, Faculty of Pharmacy, Chiang Mai University, Chiang Mai, 50200 Thailand; 3 Department of Pharmaceutical Technology, Faculty of Pharmaceutical Sciences, Huachiew Chalermprakiet University, Samutprakarn, 10250 Thailand; 4 Research Center of Pharmaceutical Nanotechnology, Chiang Mai University, Chiang Mai, 50200 Thailand; 5 Innovation Center for Holistic Health, Nutraceuticals, and Cosmeceuticals, Faculty of Pharmacy, Chiang Mai University, Chiang Mai, 50200 Thailand

**Keywords:** Biochemistry, Chemical biology

## Abstract

This study is the first to compare the chemical compositions and biological activities of a conventional dried *Dimocarpus longan* with a novel black *D. longan* that underwent a thermal ageing process. Pericarp, aril, and seed of both *D. longan* were macerated in 95% v/v ethanol. Their chemical compositions were investigated using a Folin–Ciocalteu assay, aluminum chloride assay, and high-performance liquid chromatography. Antioxidant activities were evaluated in terms of radical scavenging and iron (III) reduction capacity. An enzyme inhibition assay was used to evaluate the hyaluronidase inhibition. Inflammatory cytokine secretion was evaluated with an enzyme-linked immunosorbent assay. After being exposed to a heating and ageing procedure, gallic acid and ellagic acid content were increased tenfold, while the corilagin content was doubled. Black *D. longan* seed extract was the most potent anti-hyaluronidase and antioxidant with the strongest free radical scavenging and reduction power, while black *D. longan* aril extract resulted in the highest inhibition of inflammatory cytokine secretion. Black *D. longan* contained more biologically active compounds and possessed more potent biological activities than conventional dried *D. longan*. Therefore, thermal ageing treatment is suggested for producing black *D. longan*, for which seed extract is suggested as a cosmeceutical active ingredient and aril extract for anti-inflammation.

## Introduction

*Dimocarpus longan* Lour., a subtropical evergreen plant in the family of Sapindaceae, is widely known as longan. *D. longan* is cultivated in several countries in East Asia and South-East Asia as well as Australia and some subtropical regions in the US^1^. China and Thailand are the largest areas of commercial *D. longan* cultivation^2^. The succulent and edible aril with delicious flavor and health benefits has led to the increased popularity of *D. longan*^1^. Since the aril of *D. longan* contains several polyphenols, flavonoids, organic acids, and polysaccharides, it possesses various beneficial biological activities, including antioxidant, antiglycation, anticancer, immunomodulatory, prebiotic, anti-osteoporotic, anxiolytic, and memory-enhancing effects^3^. A decoction of dried aril has been taken as a tonic for insomnia and neurasthenic neurosis treatment since ancient times^4^. Not only does the aril of *D. longan*, which is the only edible portion, have reported beneficial effects on health, but the pericarp has also been reported to contain abundant polyphenols, flavonoids, and polysaccharides, which possess antioxidant, anti-tyrosinase, and anti-hyperglycemic activities^1^. Furthermore, *D. longan* seed, which is a waste from the food and canning industry, contains antioxidative polyphenols and possesses anti-tyrosinase, antibacterial, and anti-fungal activity^1,5–7^. *D. longan* seed has also been administered to counteract heavy sweating, whereas the ground kernel has been used for the treatment of various conditions according to their chemical components such as saponin, tannin, and fat^8^. Although *D. longan* has been reported to contain a variety of biologically active components and have the potential to be used for the treatment of various conditions, the fruit of *D. longan* has a short storage life since its pericarp rapidly turns brown and hardens at ambient temperature^1,9^. The highly perishable nature and limited shelf life of *D. longan* fruit not only decreases its marketing value after deterioration but also burdens both domestic distribution and export to foreign countries^10^. Although a low temperature (1–5 °C) can protect *D. longan* from pathological degradation, the fruit deteriorates quickly after being removed from cold storage^3^. Moreover, the over-supply of off-seasonal *D. longan* production recently led to increasing production costs and a decrease in selling prices^10^. Sopadang et al. highlighted the issues in the *D. longan* supply chain and recommended adding value to *D. longan* and creating a range of *D. longan* products as improvement alternatives to enhance *D. longan* supply chain management efficiency^10^.

Dried flesh and the canned product of *D. longan* are widely consumed and can be distributed worldwide. Generally, dried foods can be kept for a long period, but their sensory and nutritional characteristics are often lost along with the water removal during the drying processes^11^. Production of intermediate moisture food (IMF) is another technique to overcome this problem since the properties of IMF are close to fresh foods while extending shelf life^11,12^. A reduction of the moisture content and a water activity below 0.6 do not support microbial growth and lead to shelf-stable products^12,13^. Various types of food have been preserved as IMF, such as meat and several fruits, e.g., grapes, tomatoes, peaches, prunes, apricots, and strawberries^11,14^. However, some antimicrobial compounds and additives are required in the production of IMF for antimicrobial properties (e.g., preservatives, sugar, and salt), along with the agents for water activity reduction and plasticizing, e.g., glycols and sorbitol^12,13^.

Besides IMF, the heating and ageing process can also prolong the shelf life without refrigeration. A well-known food that undergoes this process is black garlic (*Allium sativum* L.), a processed garlic produced by thermal treatment of raw garlic at high temperature and high relative humidity for 60–90 days without using additives^15,16^. During the production process, raw garlic also undergoes the Maillard reaction, which occurs between amine groups and carbonyl compounds, finally resulting in brownish melanoidin^17^. Melanoidin has been shown to have a number of biological actions, including antioxidant, antibacterial, anti-inflammatory, hypoglycemic, hypotensive, and antitumor effects; prevention of obesity; lowering of serum lipopolysaccharide levels; and modulation of the composition of the gut microbiota^17,18^. Additionally, inhibition of oxidation and angiotensin I converting enzyme were enhanced in black garlic comparing to raw garlic^16^.

Therefore, the heating and ageing process not only preserves the food but also enhances its biological activities. Since the production of black *D. longan* through a heating and ageing process has not previously been reported, this study is the first to produce a novel black *D. longan* and investigate its chemical composition, as well as its potential health benefits of antioxidant, anti-inflammatory, and anti-hyaluronidase activities.

## Results and discussion

### Dried *D. longan* and black* D. longan* extracts

**Figure 1:**
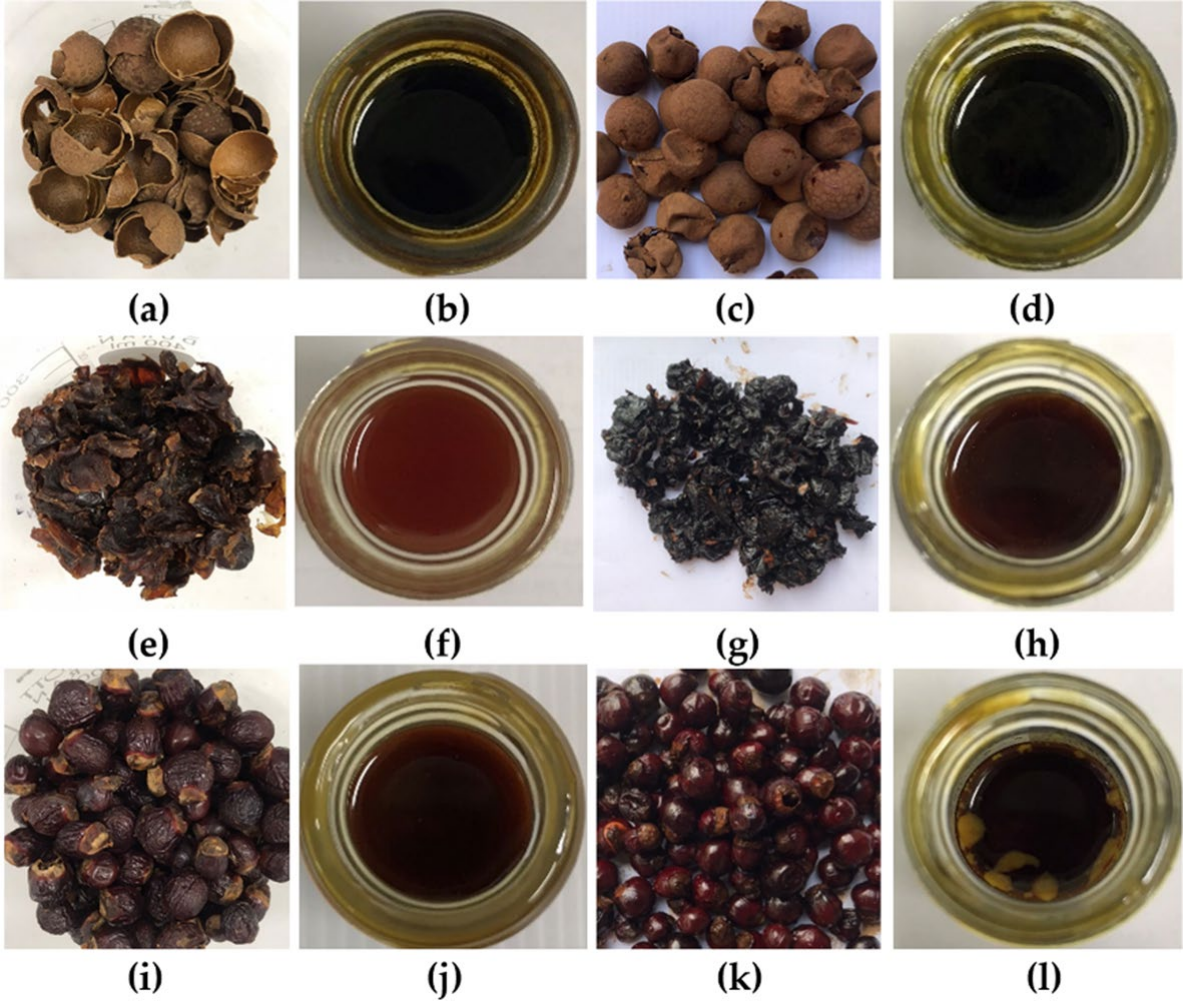
External appearance of dried *D. longan* pericarp (**a**), dried *D. longan* pericarp extract (**b**), black *D. longan* pericarp (**c**), black *D. longan* pericarp extract (**d**), dried *D. longan* aril (**e**), dried *D. longan* aril extract (**f**), black *D. longan* aril (**g**), black *D. longan* aril extract (**h**), dried *D. longan* seed (**i**), dried *D. longan* seed extract (**j**), black *D. longan* seed (**k**), and black *D. longan* seed extract (**l**).

**Figure 2:**
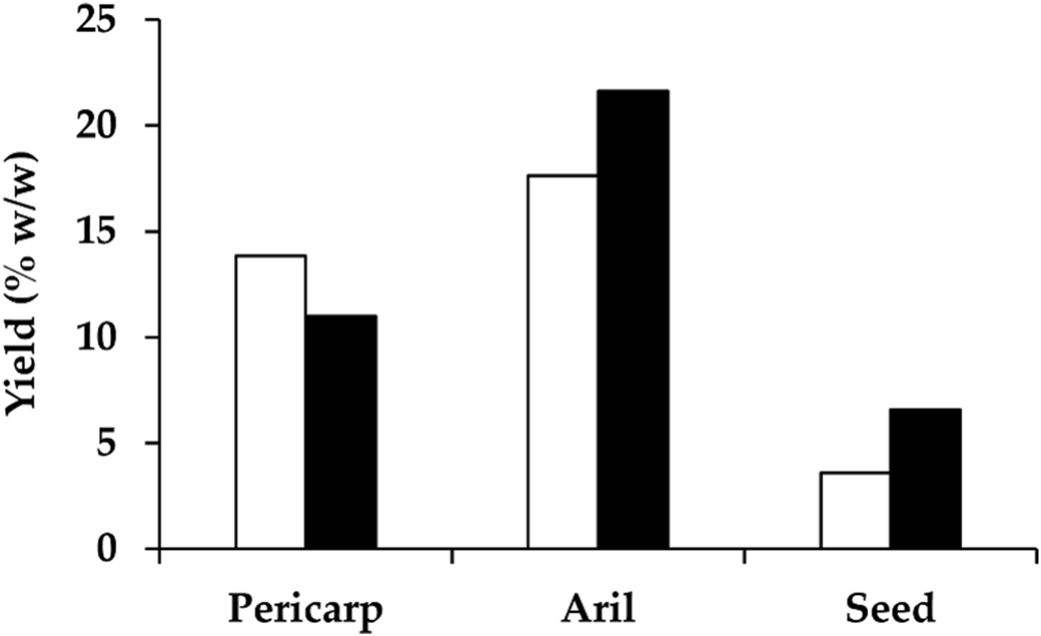
Yield of the ethanolic extracts from pericarp, aril, and seed of dried (unfilled squares) and black (filled squares) *D. longan*.

The external appearance of dried *D. longan* is different from black *D. longan* as shown in Fig. 1. The color of pericarp, aril, and seed of black *D. longan* was obviously darker than that from dried *D. longan*, especially the aril, which turned from dark brownish color to black. The pericarp and seed of black *D. longan* were substantially more moist than dried *D. longan*. The outer part of dried *D. longan* seed was shriveled, whereas the dried *D. longan* pericarp was dry and brittle. All *D. longan* extracts were of semisolid mass with different color as shown in Fig. 1. The color of black *D. longan* extracts was darker than the dried *D. longan* extracts. The color of pericarp extracts was the darkest, followed by the extract from seed and aril. Yields of each *D. longan* extract are shown in Fig. 2. The aril yielded the highest extract content, followed by pericarp and seed. The highest yield of the extract was obtained from black *D. longan* aril (21.6% w/w), followed by dried *D. longan* aril (17.6% w/w), dried *D. longan* pericarp (13.8% w/w), black *D. longan* aril (11.0% w/w), black *D. longan* seed (6.6% w/w), and dried *D. longan* seed (3.6% w/w). It was highlighted that black *D. longan* yielded higher extract content compared to dried *D. longan* in aril and seed but not in pericarp. The explanation is that the pericarp of dried *D. longan* lost more water content than that of black *D. longan*, which underwent a heating and ageing process in conditions that kept the relative humidity constant at 75%. Therefore, the mass of initial pericarp material of dried *D. longan* was lower and led to a higher extract yield since the yield was calculated from the initial pericarp material used in the extraction process.

### Chemical compositions of dried *D. longan* and black* D. longan* extracts

**Figure 3:**
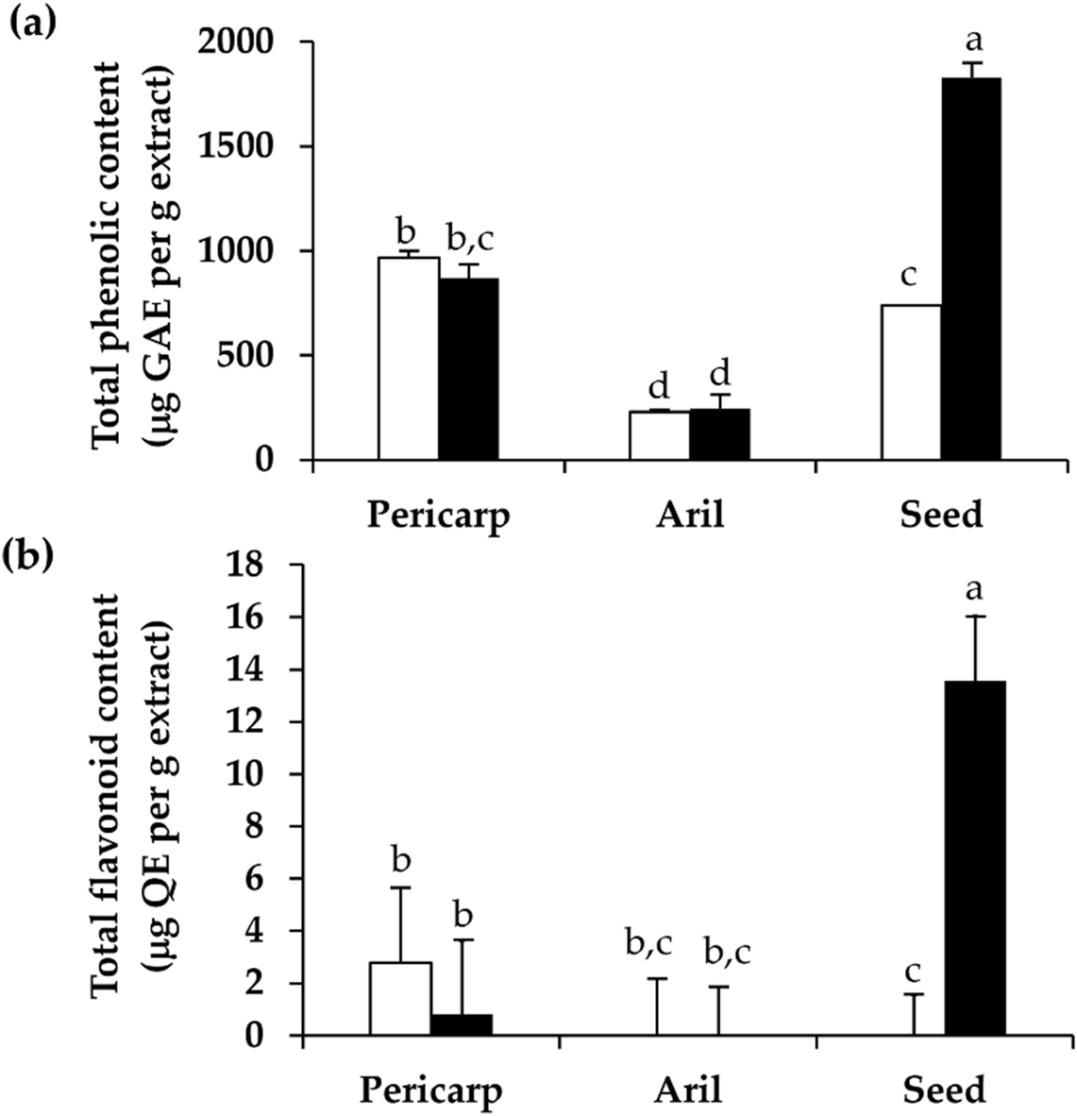
Total phenolic content (**a**) and total flavonoid content (**b)** of the ethanolic extracts from pericarp, aril, and seed of dried (unfilled squares) and black (filled squares) *D. longan*. The letters (a, b, c, and d) denote significant differences in total phenolic content or total flavonoid content among various *D. longan* extracts (p < 0.05) when analyzed using one-way analysis of variance (ANOVA) followed by Tukey's post-hoc tests (n = 3).

*Dimocarpus longan* extracts were investigated for contents of total phenolic compounds, total flavonoid, gallic acid, corilagin, and ellagic acid. Gallic acid and corilagin are natural polyphenolic compounds which belong to hydrolysable tannin, whereas ellagic acid belongs to a flavonoid group^19,20^. Among different parts of dried *D. longan*, pericarp extracts contained the significantly highest total phenolic content (p < 0.05) and the highest total flavonoids as shown in Fig. 3. The results agreed well with a previous study, which reported that polyphenolic compounds are abundant in pericarp and seed of *D. longan* compared to the *D. longan* aril^1^. The total phenolic content of pericarp, seed, and aril extracts from dried *D. longan*, which were 967.6 ± 31.5, 739.3 ± 62.3, and 229.5 ± 2.6 µg GAE per g extracts, were found to be in agreement with a previous study, which reported that the total phenolic content of *D. longan* was in the range of 22.09–132.47 mg gallic acid equivalent (GAE/100 g), which was equivalent to 220.9–1324.7 µg GAE per g extract^1^. Interestingly, the dramatic increase in total phenolic content was observed in black *D. longan* seed extract. The ethanolic extract from black *D. longan* seed contained as much as 1827.1 ± 73.1 µg GAE per g extracts, which was much higher than previously reported^1^. On the other hand, there was no significant difference between the total phenolic content of dried and black *D. longan* extract from pericarp and aril (p > 0.05).

In addition to the total phenolic content, black *D. longan* seed extract also contained the significantly highest flavonoid contents (p < 0.05). Among various dried *D. longan* extracts, the pericarp contained the significantly highest flavonoid content of 2.8 ± 2.4 µg QE per g extract as shown in Fig. 3 (p < 0.05). The results were in accordance with a previous study, which reported that the quercetin content of *D. longan* pericarp was 3.12 ± 0.76 mg/kg, which was equivalent to 3.12 ± 0.76 µg/g extract^21^. *D. longan* pericarp has been reported to contain slightly higher content of flavonoids than *D. longan* seed and aril^22^. Obviously, the flavonoid content of black *D. longan* seed extract, which was as high as 13.6 ± 2.5 µg QE per g extract, was dramatically enhanced and was about four times higher than previously reported.

Although the thermal ageing process of *D. longan* did not affect the phenolic and flavonoid contents of *D. longan* pericarp and aril, the total phenolic and flavonoid contents of *D. longan* seed were obviously enhanced after the production process of black *D. longan*. The likely explanation might be the formation of biological compounds, which were not originally present in the *D. longan* seed, during the thermal ageing process such as resistant starch^23,24^. During the temperature/time-controlled incubation, the starch inclusion complexes were generated by interaction with other components in the seeds^24^.

**Figure 4:**
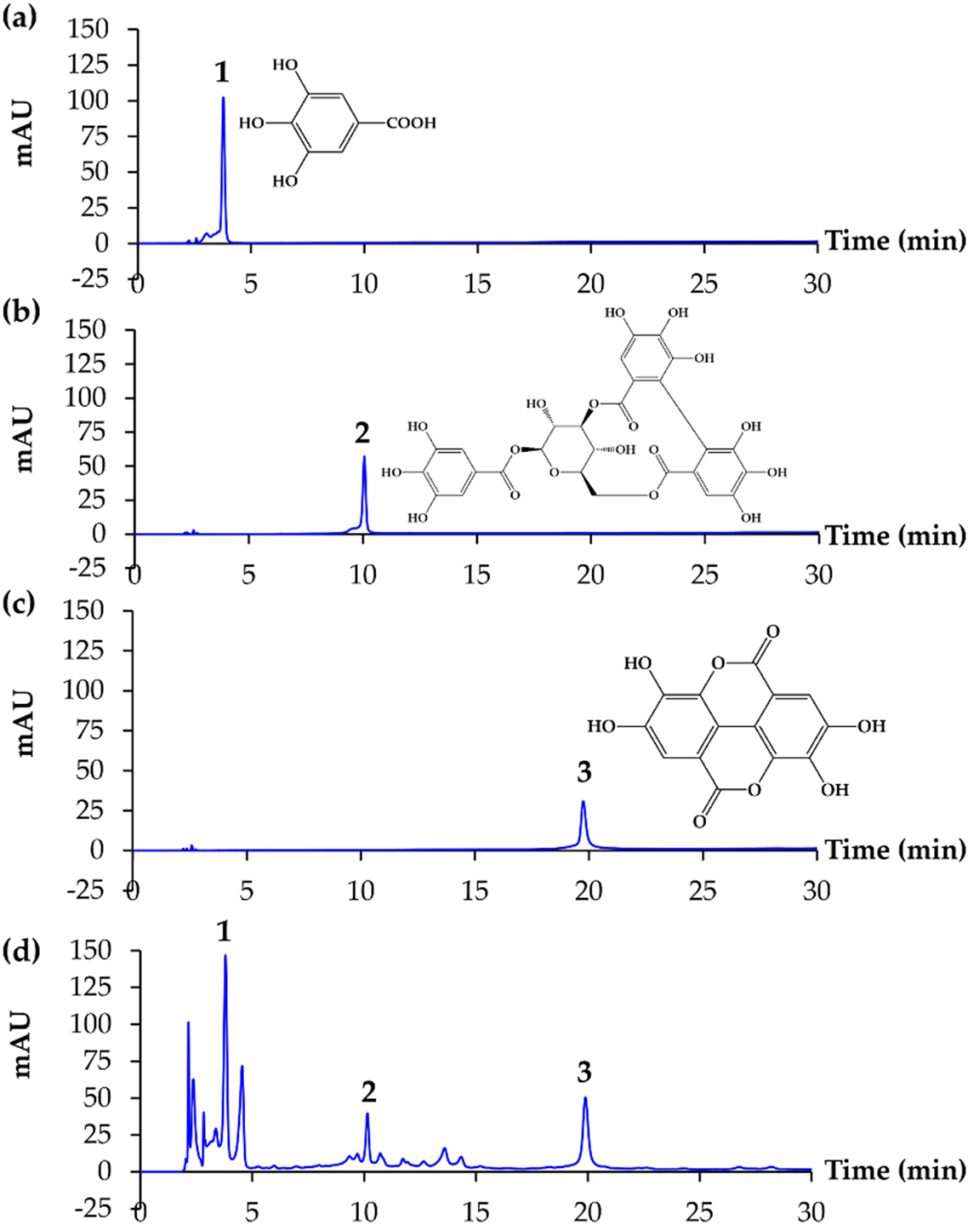
HPLC chromatograms of gallic acid (**a**), corilagin (**b**), ellagic acid (**c**), and black *D. longan* seed extract (**d**).

As shown in Fig. 4, gallic acid (1), corilagin (2), and ellagic acid (3) were detected in *D. longan* extract. The peak of gallic acid, corilagin, and ellagic acid in the HPLC chromatograms were detected at around 3.7, 9.9, and 19.2 min, respectively. The results agreed with the previous studies, which identified these compounds as major polyphenolic components of *D. longan* pericarp and seed^25–27^.

**Figure 5:**
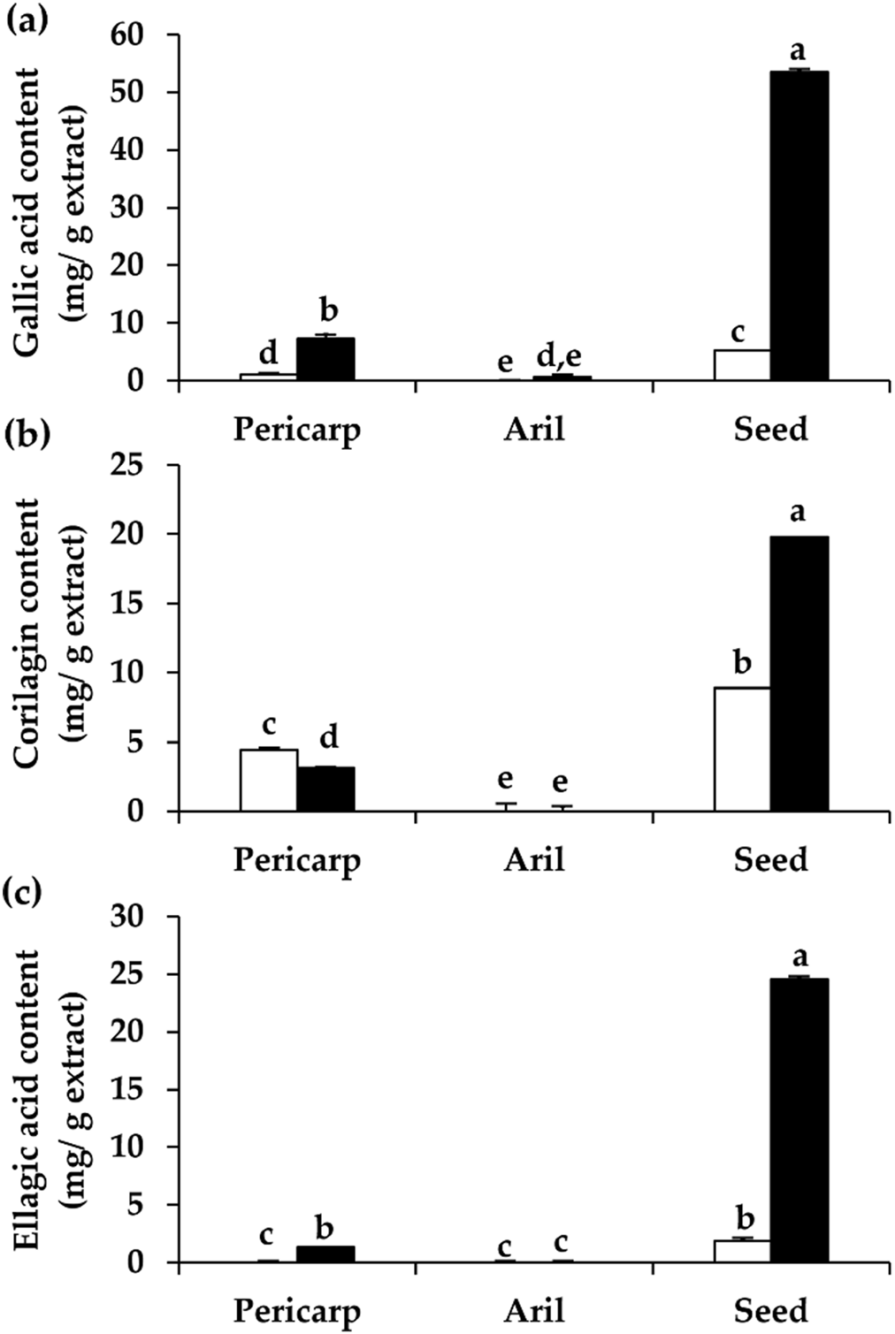
Gallic acid content (**a**), corilagin content (**b**), and ellagic acid content (**c**) of the ethanolic extracts from pericarp, aril, and seed of dried (unfilled squares) and black (filled squares) *D. longan*. The letters (a, b, c, d, and e) denote significant differences in the contents of gallic acid, corilagin, or ellagic acid among various *D. longan* extracts (p < 0.05) when analyzed using one-way analysis of variance (ANOVA) followed by Tukey’s post-hoc tests (n = 3).

In the present study, the contents of these polyphenolic components were investigated in the context of a comparison between black *D. longan* extract and dried *D. longan* extracts. The amounts of each compound in *D. longan* extracts are shown in Fig. 5. The findings were in line with the total phenolic and total flavonoid contents since black *D. longan* seed extract contained the significantly highest quantities of polyphenolic compounds and flavonoid contents (p < 0.05).

Among various parts of dried *D. longan* fruit, seeds contained the significantly highest content of gallic acid, corilagin, and ellagic acid (p < 0.05) with 5.3 ± 0.0, 8.9 ± 0.1, and 1.9 ± 0.2 mg/g extract, respectively. Interestingly, the these phenolic and flavonoid contents were significantly enhanced after the production process of black *D. longan* (p < 0.05). The gallic acid, corilagin, and ellagic acid content of black *D. longan* seed extract were as high as 53.6 ± 0.9, 19.8 ± 2.9, and 24.5 ± 0.7 mg/g extract, respectively. After being exposed to a heating and ageing procedure, the amount of gallic acid and ellagic acid of *D. longan* were increased by around 10-fold, while the quantity of corilagin was doubled. The reason might be the liberation of free polyphenolic compounds and flavonoids from the bound forms (i.e., esterified and glycosylate) or the decline in enzymatic oxidation involving in the antioxidant compounds in the raw fruit^28^. The results of black *D. longan* were in accordance with those of black garlic, as the total phenolic and total flavonoid contents of the garlic subjected to the thermal processing steps were significantly higher than those of fresh garlic^23,28^. The previous study reported that the phenolic content was increased by about 4–10-fold in the black garlic cloves compared with the fresh garlic^23^.

Apart from the findings showing differing content of the biologically active component in various parts of *D. longan* fruit, different methods used in the drying process also affected their bioactive compounds. The thermal ageing process was hence proposed for the enhancement of bioactive compounds in *D. longan.*

### Antioxidant activities of dried *D. longan* and black* D. longan* extracts

**Figure 6:**
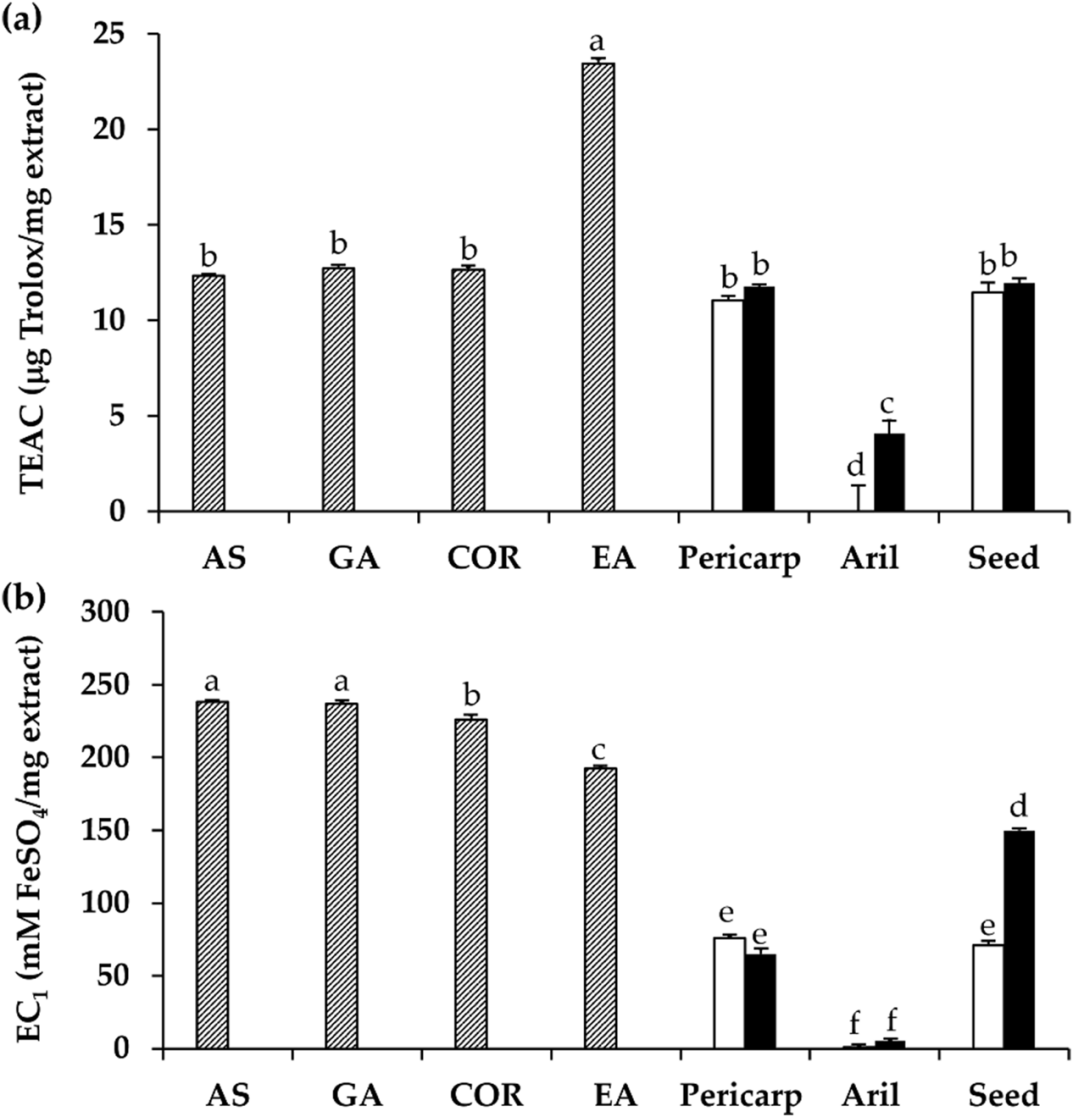
Trolox equivalent antioxidant capacity (TEAC) (**a**) and equivalent concentration (EC_1_) (**b**) of ascorbic acid (AS), gallic acid (GA), corilagin (CO), ellagic acid (EA), and the ethanolic extracts from pericarp, aril, and seed of dried (unfilled squares) and black (filled squares) *D. longan*. The letters (a, b, c, d, e, and f) denote significant differences in TEAC or EC_1_ values among various tested samples (p < 0.05) when analyzed using one-way analysis of variance (ANOVA) followed by Tukey’s post-hoc tests (n = 3).

The antioxidant activities of dried and black *D. longan* extracts were investigated by two assays with different mechanisms of action. The ABTS assays measure the electron transfer reaction and represent the radical scavenging activity of the tested samples, while the FRAP assay is concerned with the ion reduction process, which represents the ability of the tested compound to convert ferric ions (Fe^3+^) to ferrous ions (Fe^2+^)^29^. The ferric reducing antioxidant power (EC_1_) and Trolox equivalent antioxidant capacity (TEAC) values of dried and black *D. longan* extracts are shown in Fig. 6.

The TEAC values of black *D. longan* extracts were not significantly different from those for the dried *D. longan* extracts, except in the aril. The dried *D. longan* aril extract had no antioxidant activity, whereas the black *D. longan* aril extract possessed some antioxidant activity with a TEAC value of 4.1 ± 1.4 µg Trolox/mg extract. A probable explanation lies in the greater Maillard reaction, which occurs in the aril as compared with the others. As *D. longan* aril is composed of glucose, fructose, and various types of amino acids, such as γ-aminobutyric acid, it tends to undergo Maillard reactions, which are the chemical reactions between an amino acid and a reducing sugar that occur in the presence of heat^30^. These non‐enzymatic browning reactions gave black *D. longan* a darker color and resulted in the formation of some antioxidant compounds^28^.

On the other hand, black *D. longan* pericarp and seed extracts possessed the same radical scavenging activity as those from dried *D. longan*. A likely explanation might be the degradation of some oxidative compounds during the heating process, although some free polyphenolic compounds and flavonoids were liberated from the bound forms^23^. Interestingly, the TEAC values of pericarp and seed extracts from both dried and black *D. longan* were comparable to ascorbic acid, gallic acid, and corilagin (p > 0.05). Ellagic acid was remarked as the most potent radical scavenger (TEAC = 23.4 ± 0.3 µg Trolox/mg), followed by ascorbic acid (TEAC = 12.3 ± 0.0 µg Trolox/mg), gallic acid (TEAC = 12.8 ± 0.2 µg Trolox/mg), and corilagin (TEAC = 12.7 ± 0.1 µg Trolox/mg). Thereby, ellagic acid was found to be the main compound responsible for the free radical scavenging activity of *D. longan* extracts together with gallic acid and corilagin^31^. Although the previous study reported that among various polyphenolic compounds, tannins demonstrated the strongest ABTS·+ radical scavenging activity^32^, in the present study it was observed that ellagic acid, which belongs to a flavonoid group, was more potent than gallic acid and corilagin, which belongs to hydrolysable tannin^1^. Furthermore, *D. longan* extracts from both pericarp and seed could therefore be considered as natural extracts with potent radical scavenging activity.

Aside from radical scavenging activity, *D. longan* extracts also possessed a reduction ability as shown in Fig. 6. The reduction ability of *D. longan* extracts was in accordance with their phenolic and flavonoid contents. Gallic acid possessed the significantly highest EC_1_ value of 237.0 ± 1.6 mM FeSO_4_/mg, which was comparable to that of ascorbic acid (238.3 ± 0.2 mM FeSO_4_/mg), followed by corilagin (226.2 ± 2.9 mM FeSO_4_/mg) and ellagic acid (192.3 ± 0.7 mM FeSO_4_/mg). However, both phenolic compounds and flavonoids were responsible for their reduction capacity^33^. The black *D. longan* seed extract, which contained the highest total phenolic, total flavonoid, gallic acid, corilagin, and ellagic acid contents, thus possessed the significantly highest reduction ability with an EC_1_ value of 150.0 ± 1.0 mM FeSO_4_/mg extract (p < 0.05). Consequently, the black *D. longan* seed extract was suggested as the most potent antioxidant extract with the strongest free radical scavenging and reduction ability. Because of its potent antioxidant effect, black *D. longan* seed has been proposed as a natural antioxidant source for use in food and cosmetic products. Since the portions of pericarp and seed account for 30% of the whole fruit dry weight^34^, the utilization of these by-products would not only reduce the agricultural waste product but also increase its value.

### Anti-inflammatory activities of dried *D. longan* and black* D. longan* extracts

The inhibitory activities against the secretion of IL-6 and TNF-α, which are key players involved in the age-related inflammatory process^35^, of dried and black *D. longan* extracts were investigated. RAW 264.7 macrophage cells were used in the present study since they can secret these cytokines after the stimulation of LPS. The RAW 264.7 macrophage cell viability after treatment with dried and black *D. longan* extracts is shown in Table 1. No cytotoxicity was detected in any of the *D. longan* extracts since the cell viability was more than 100%. Dexamethasone, corilagin, gallic acid, and ellagic acid were also found to be safe for RAW 264.7 macrophage cells.

**Table 1:** RAW 264.7 macrophage cell viability.

Samples	Cell viability (%)
Dexamethasone	98.9 ± 3.8
Corilagin	102.0 ± 0.2
Gallic acid	102.0 ± 0.2
Ellagic acid	105.4 ± 1.8
**Dried *D. longan***	
Aril	102.8 ± 0.1
Pericarp	101.3 ± 0.2
Seed	102.6 ± 1.3
**Black *D. longan***	
Aril	103.0 ± 0.3
Pericarp	103.1 ± 0.9
Seed	103.0 ± 0.3

The final concentration of each sample was 1 µg/mL.

**Figure 7:**
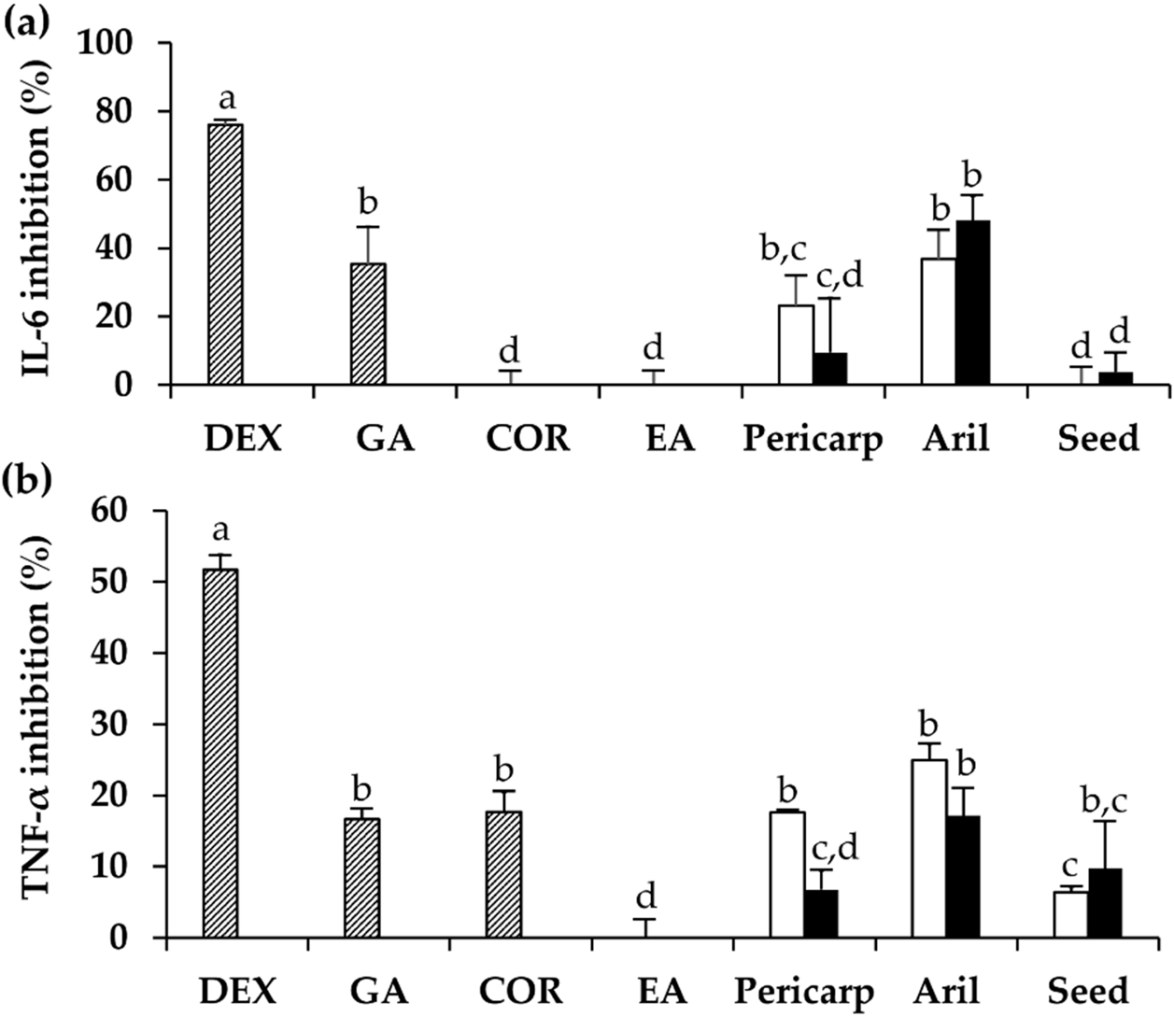
Inhibitory activities against the secretion of interleukin-6 (IL-6) (**a**) and tumor necrosis factor-α (TNF-α) (**b**) of dexamethasone (DEX), gallic acid (GA), corilagin (CO), ellagic acid (EA), and the ethanolic extracts from pericarp, aril, and seed of dried (unfilled squares) and black (filled squares) *D. longan*. The final concentration of each sample was 1 µg/mL. The letters (a, b, c, and d) denote significant differences in IL-6 or TNF-α inhibition among various tested samples (p < 0.05) when analyzed using one-way analysis of variance (ANOVA) followed by Tukey's post-hoc tests (n = 3).

The IL-6 and TNF-α inhibitory activities of dried and black *D. longan* extracts are shown in Fig. 7. TNF-α is known as an indicator of chronic inflammatory processes related to ageing, whereas IL-6 has been associated with poor physical performance and muscle weakness by geriatricians and could predict the onset of disability^36,37^. Among various parts of *D. longan* fruit, aril of both dried and black *D. longan* was predominant in IL-6 and TNF-α inhibition. Gallic acid was suggested to be the main compound responsible for both IL-6 and TNF-α inhibitory activities. In contrast, corilagin was responsible only for TNF-α inhibition. Although *D. longan* extracts and their major chemical components exhibited only low to moderate anti-inflammatory activities compared to dexamethasone, a corticosteroid used in the treatment of inflammation, they could be consumed as natural anti-inflammatory supplements with no steroidal side effects.

### Anti-hyaluronidase activities of dried *D. longan* and black* D. longan* extracts

Hyaluronidase, a homologous enzyme that hydrolyzes or depolymerizes hyaluronan, plays an important role in the modulating activity of many pathological processes^38^. Hyaluronan plays a pivotal role in the maintenance of the elastoviscosity of liquid connective tissues and controls the water transportation related to the tissue hydration^39^. The degradation of hyaluronan resulting in the production of breakdown products, including lower molecular mass polymers. These breakdown products of hyaluronan exhibited distinct biological properties from the larger precursor molecules^40^. The hyaluronan depolymerization occurs in tissue injury and initiates the inflammatory response^38^. Additionally, hyaluronan is known as a lubricant and shock-absorber in joints and connective tissues^41^. Its degradation hence leads to the deterioration of the viscoelastic properties of the synovial fluid^42^.

**Figure 8:**
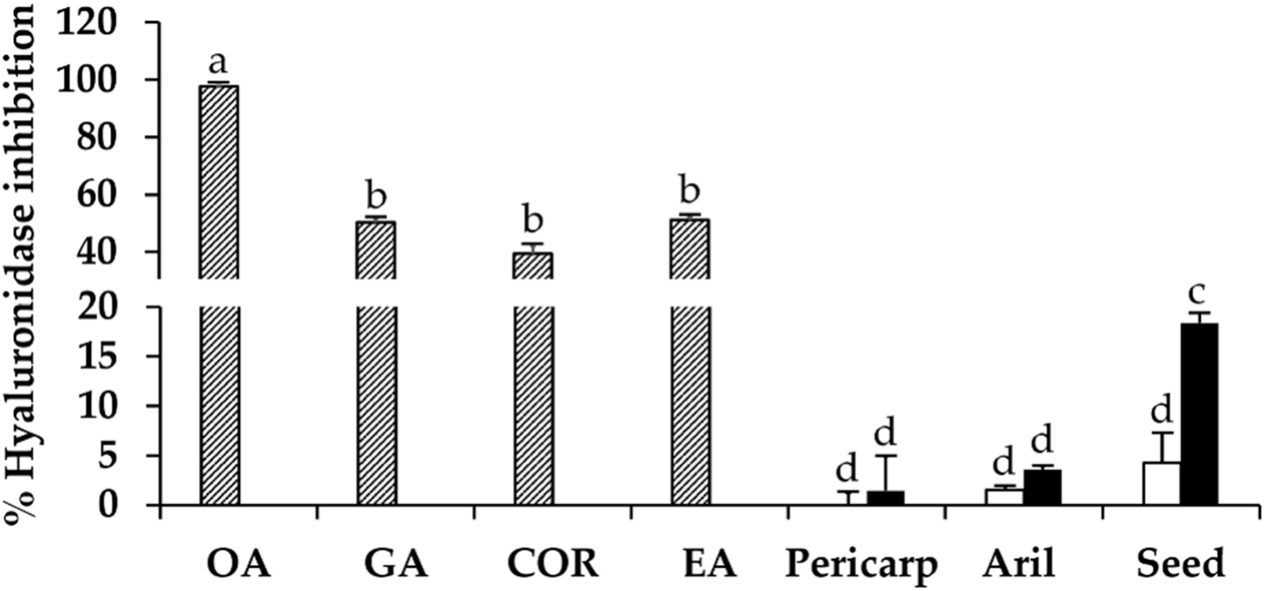
Inhibition against hyaluronidase activity of oleanolic acid (OA), gallic acid (GA), corilagin (CO), ellagic acid (EA), and the ethanolic extracts from pericarp, aril, and seed of dried (unfilled squares) and black (filled squares) *D. longan*. The letters (a, b, c, and d) denote significant differences in hyaluronidase inhibition among various tested samples (p < 0.05) when analyzed using one-way analysis of variance (ANOVA) followed by Tukey's post-hoc tests (n = 3).

The inhibitory activities against hyaluronidase of dried and black *D. longan* extracts are shown in Fig. 8. Although *D. longan* extracts exhibited low anti-hyaluronidase activity, the inhibitory effect of black *D. longan* seed was significantly enhanced compared to the dried *D. longan* seed extract. Since the anti-hyaluronidase activity of black *D. longan* seed extract (18.4 ± 2.0%) was the most significantly potent (p < 0.05), black *D. longan* seed extract could be suggested to have anti-hyaluronidase activity in addition to its antioxidant activities.

In conclusion, black *D. longan* was successfully developed after undergoing a heating and ageing procedure at a controlled temperature of 70 °C and a relative humidity of 75%. A novel black *D. longan* contained a larger quantity of biologically active compounds and possessed more potent biological activities than a conventional dried *D. longan*. The ethanolic extract from the seed of black *D. longan* contained the most significantly abundant of biologically active compounds, including total phenolic, total flavonoid, gallic acid, corilagin, and ellagic acid content (p < 0.05). Furthermore, it possessed the most significantly potent antioxidant and anti-hyaluronidase activities (p < 0.05). Since oxidative stress is known to be related to ageing and skin wrinkles, black *D. longan* seed extract with a potent antioxidant activity (p < 0.05) was suggested for further topical use as a cosmeceutical ingredient for anti-skin ageing. On the other hand, degradation of hyaluronan in the skin resulted in the loss of the skin’s natural moisturizing factor, while the loss of hyaluronan from the synovial fluid in joint resulted in joint pain and several conditions. Therefore, black *D. longan* seed extract, which significantly inhibited hyaluronidase activity (p < 0.05), is suggested for both topical use for anti-skin ageing and joint pain relief. On the other hand, the aril of *D. longan*, which possessed the significantly highest anti-inflammatory activities, is suggested as a natural edible anti-inflammatory agent.

## Material and methods

### Chemical material

l-Ascorbic acid, aluminum chloride (AlCl_3_), 2,2′-azino-bis3-ethylbenzothiazoline-6-sulfonic acid (ABTS), calcium chloride (CaCl_2_), corilagin, dexamethasone, disodium phosphate (Na_2_HPO_4_), ferric chloride (FeCl_3_), ferrous chloride (FeCl_2_), ferrous sulfate (FeSO_4_), formic acid, Folin–Ciocalteu reagent, 4,4′,5,5′,6,6′-hexahydroxy-diphenic acid 2,6,2′,6′-dilactone (ellagic acid), hyaluronic acid, hydrochloric acid (HCl), 6-hydroxy-2,5,7,8-tetramethylchroman-2-carboxylic acid (Trolox), bovine testicular hyaluronidase (E.C.3.2.1.3.5), potassium acetate (CH_3_COOK), potassium persulphate (K_2_S_2_O_8_), sodium acetate (C_2_H_3_NaO_2_), sodium carbonate (Na_2_CO_3_), sodium chloride (NaCl), sodium dihydrogen phosphate (NaH_2_PO_4_), sodium phosphate (Na_3_PO_4_), 2,4,6-tripyridyl-striazine (TPTZ), and 3,4,5-trihydroxybenzoic acid (gallic acid) were analytical grade and purchased from Sigma-Aldrich (St. Louis, MO, USA). Amphotericin B, Dulbecco's modified Eagle's medium, high glucose (DME/HIGH), l-glutamine, penicillin/streptomycin, trypan blue, and secondary antibody conjugated with HRP were bought from Invitrogen (Carlsbad, CA, USA). Lipopolysaccharides (LPS) were bought from Cell Signaling Technology (Danvers, MA, USA). GlutaMAX-I supplement was bought from Thermo Fisher Scientific, Inc. (Thermo Fisher Scientific, Waltham, MA, USA). Newborn bovine calf serum (catalog number: 16010159), fetal bovine serum (FBS) (catalog number: 26140079), and bovine serum albumin (BSA) (catalog number: B14) were bought from Gibco (Thermo Fisher Scientific, Waltham, MA, USA). Analytical grade acetic acid, ethanol, and dimethyl sulfoxide (DMSO) were purchased from Labscan, Ltd. (Dublin, Ireland). HPLC-grade acetonitrile was purchased from Labscan, Ltd. (Dublin, Ireland).

### Plant material

The *D. longan* fruits were collected from Chiang Mai Province in Northern Thailand in January 2020 by the gardeners according to WHO Guidelines on Good Agricultural and Collection Practices (GACP) for Medicinal Plants^43^. The permission to collect *D. longan* fruits were obtained from the farm owner. The plant materials were identified and authenticated by Ms. Wannaree Charoensup, a botanist at the Herbarium of Faculty of Pharmacy, Chiang Mai University. A voucher specimen number 0023268 of *D. longan* has been deposited in an Herbarium, Department of Pharmaceutical Science, Faculty of Pharmacy, Chiang Mai University. The preparation of conventional dried and black *D. longan* was performed by the Faculty of Agro-Industry, Chiang Mai University, Chiang Mai, Thailand.

### Conventional dried *D. longan* preparation

Conventional dried *D. longan* was obtained after the whole fruit of fresh *D. longan* was incubated in an oven set at the temperature of 50 °C until dryness (moisture content below 16–18%). The sample of dried *D. longan* was kept in a sealed plastic bag to prevent contact with air and humidity in the room temperature until further experiments.

### Black *D. longan* preparation by thermal and ageing process

Black *D. longan* was obtained after the whole fruit of dried *D. longan* was incubated for 20 days at a controlled temperature of 70 °C and 75% relative humidity. The sample of black *D. longan* was kept in a sealed plastic bag and placed in a desiccator chamber cabinet to prevent contact with air and humidity at room temperature until further experiments.

### Preparation of dried *D. longan* and black* D. longan* extracts

The seed, aril, and pericarp were separated from each other. Each part of the *D. longan* fruit was ground into fine powder using a 20-in. herbal medicine grinder tub with a powerful motor (Thai Pradith Industry Co., Ltd., Bangkok, Thailand). Dried *D. longan* powder was subsequently macerated in 95% v/v ethanol for three cycles of 24 h. The proportion of plant material to solvent was 1:5 by weight. The same protocol of maceration was used for each part of dried and black *D. longan*. The extracting solvent from three cycles was combined and removed using a rotary evaporator (Buchi Labortechnik GmbH, Essen, Germany). All extracts were stored in the refrigerator ($\sim$ 4 °C) until further experiments.

### Determination of phytochemical compositions of dried *D. longan* and black* D. longan* extracts

#### Total phenolic content determination

Each *D. longan* extract was analyzed for total phenolic content using the Folin–Ciocalteu method according to the previously described method^44^. Firstly, 20 μL of the ethanolic solution of *D. longan* extracts (1 mg/mL) was mixed with 180 μL of Folin–Ciocalteu reagent (10% w/v). After incubation at ambient temperature for 4 min, 80 μL of sodium carbonate solution (74.2 g/L, 0.7 M) was added. After the resulting mixtures were incubated for 2 h. they were measured for an absorbance at 750 nm using multimode detector (SPECTROstar Nano, BMG Labtech, Offenburg, Germany). The results were presented in the form of gallic acid equivalent values (GAE) representing an amount of gallic acid (µg) per g of the *D. longan* extracts. GAE was calculated following Eq. (1);

1 $${\text{X}} = {{\left( {{\text{Y}} - 0.00{75}} \right)} \mathord{\left/ {\vphantom {{\left( {{\text{Y}} - 0.00{75}} \right)} {0.{3812}\left( {{\text{R}}^{{2}} = 0.{9985}} \right)}}} \right. \kern-\nulldelimiterspace} {0.{3812}\left( {{\text{R}}^{{2}} = 0.{9985}} \right)}},$$

where X is GAE or µg of gallic acid per g of the *D. longan* extracts and Y is an absorbance of each sample tested with the Folin–Ciocalteu assay. The experiments were performed in triplicate.

#### Total flavonoid content determination

Total flavonoid content of each *D. longan* extract was investigated using the aluminum chloride method, which has been previously described, with some modifications^45^. Firstly, 20 μL of the ethanolic solution of *D. longan* extracts (1 mg/mL) was mixed with 80 μL of AlCl_3_ aqueous solution (0.1 g/mL, 10% w/v) and 100 μL of CH_3_COOK aqueous solution (98.15 g/l, 1 M). After the resulting mixtures were incubated for 30 min in the dark, they were measured for an absorbance at 415 nm using a multimode detector (SPECTROstar Nano, BMG Labtech, Offenburg, Germany). Quercetin was applied as a standard compound to construct a calibration curve. Finally, the results were presented as quercetin equivalent (QE) values, which represented a µg of quercetin per g of the *D. longan* extracts. QE was calculated following Eq. (2);

2 $${\text{X}} = {{\left( {{\text{Y}} + 0.0{33}} \right)} \mathord{\left/ {\vphantom {{\left( {{\text{Y}} + 0.0{33}} \right)} {0.{1}0{7}\left( {{\text{R}}^{{2}} = 0.{9933}} \right)}}} \right. \kern-\nulldelimiterspace} {0.{1}0{7}\left( {{\text{R}}^{{2}} = 0.{9933}} \right)}},$$

where X is QE or µg of quercetin per g of the *D. longan* extracts and Y is an absorbance of each sample tested in the aluminum chloride assay. The experiments were performed in triplicate.

#### Determination of gallic acid, corilagin, and ellagic acid content by high performance liquid chromatography (HPLC)

The quantitative analysis of gallic acid, corilagin, and ellagic acid was performed using an HP 1100 chromatographic system (Hewlett-Packard, Waldbronn, Germany). A gradient mobile phase system composed of two phases was used, including phase A (0.05% formic acid in acetonitrile) and phase B (0.05% formic acid aqueous solution). The program was set for gradient elution of 10% A (0–8 min), 20% A (8–28 min), 30% A (28–30 min), and 10% A (30–35 min), eluting the sample at a flow rate of 1.0 mL/min. The UV detector was set at 280 nm with a Eurospher II 100-5 C18 (250 × 4.6 mm, i.d. 5 µm, Knauer, Berlin, Germany). All samples, standard solution, and mobile phase were filtrated through a 0.45 mm Millipore filter, type GV (Millipore, Bedford, MA) before injection into the HPLC system. The injected volume was set at 20 μL. The sample of *D. longan* extracts was prepared at a concentration of 1 mg/mL. Various concentrations of standard gallic acid (10–150 µg/mL), ellagic acid (5–100 µg/mL), and corilagin (2–80 µg/mL) solution were used for the construction of standard curves for quantitative determination. Subsequently, the content of gallic acid, corilagin, and ellagic acid was then calculated using the following Eqs. (3)–(5), respectively

3 $${\text{X1}} = {{\left( {{1}00{\text{A}} + {1296}} \right)} \mathord{\left/ {\vphantom {{\left( {{1}00{\text{A}} + {1296}} \right)} {{26}.{\text{8C}}\left( {{\text{R}}^{{2}} = 0.{9964}} \right)}}} \right. \kern-\nulldelimiterspace} {{26}.{\text{8C}}\left( {{\text{R}}^{{2}} = 0.{9964}} \right)}},$$

where X1 is the gallic acid concentration, A is the area under the curve (AUC) of the gallic acid peak detected around 4 min, and C is the concentration of the respective sample solution.

4 $${\text{X2}} = {{\left( {{1}00{\text{A}} + {2325}} \right)} \mathord{\left/ {\vphantom {{\left( {{1}00{\text{A}} + {2325}} \right)} {{17}.{\text{8C}}\left( {{\text{R}}^{{2}} = 0.{9996}} \right)}}} \right. \kern-\nulldelimiterspace} {{17}.{\text{8C}}\left( {{\text{R}}^{{2}} = 0.{9996}} \right)}},$$

where X2 is the concentration of corilagin, A is the AUC of the corilagin peak detected around 10 min, and C is the concentration of the respective sample solution.

5 $${\text{X3}} = {{\left( {{1}00{\text{A}} + {13},{372}} \right)} \mathord{\left/ {\vphantom {{\left( {{1}00{\text{A}} + {13},{372}} \right)} {{33}.{\text{7C}}\left( {{\text{R}}^{{2}} = 0.{9957}} \right)}}} \right. \kern-\nulldelimiterspace} {{33}.{\text{7C}}\left( {{\text{R}}^{{2}} = 0.{9957}} \right)}},$$

where X3 is the concentration of ellagic acid, A is the AUC of the ellagic acid peak detected around 20 min, and C is the concentration of the respective sample solution.

### Antioxidant activity determination of dried *D. longan* and black* D. longan* extracts with 2,2′-azinobis (3-ethylbenzothiazoline-6-sulfonic acid) (ABTS) assay

The radical scavenging effects against ABTS·^+^ of *D. longan* extracts, gallic acid, corilagin, and ellagic acid were evaluated using the ABTS assay and reported in terms of Trolox equivalent antioxidant capacity (TEAC), which represents the quantity of Trolox that equivalent to 1 mg of the *D. longa*n extracts^46^. Firstly, 20 μL of the ethanolic solution of *D. longan* extracts (1 mg/mL) was mixed with the mixture of 72 μL ABTS solution (3.60 g/L, 7.0 mM) and 108 μL potassium persulfate solution (0.66 g/L, 2.45 mM), which had been previously mixed and incubated in the dark for 16 h at ambient temperature. After the resulting mixtures were incubated for 5 min, they were measured for an absorbance at 750 nm using multimode detector (SPECTROstar Nano, BMG Labtech, Offenburg, Germany). TEAC values were calculated following Eq. (6);

6 $${\text{X}} = {{\left( {{\text{Y}} - {1}.{2}0{28}} \right)} \mathord{\left/ {\vphantom {{\left( {{\text{Y}} - {1}.{2}0{28}} \right)} {{7}.{9964}\left( {{\text{R}}^{{2}} = 0.{9977}} \right)}}} \right. \kern-\nulldelimiterspace} {{7}.{9964}\left( {{\text{R}}^{{2}} = 0.{9977}} \right)}},$$

where X is TEAC value and Y is an absorbance of each sample tested in ABTS assay. l-Ascorbic acid was used as a positive control. The experiments were performed in triplicate.

#### Ferric reduction/antioxidant power (FRAP) assay

The reduction capacity of *D. longan* extracts, gallic acid, corilagin, and ellagic acid were investigated by means of a ferric ion reduction assay^44^. Firstly, 20 μL of the ethanolic solution of *D. longan* extracts (1 mg/mL) was mixed with 150 μL acetate buffer pH 3.6 (0.3 M), 15 μL TPTZ solution (3.123 g/L, 10 mM) in HCl (14.58 g/L, 40 mM), and freshly prepared 15 μL FeCl_3_ (3.24 g/L, 20 mM). After incubation at ambient temperature for 4 min, 80 μL of sodium carbonate solution (74.2 g/L, 0.7 M) was added. After the resulting mixtures were incubated for 5 min, they were measured for an absorbance at 595 nm using multimode detector (SPECTROstar Nano, BMG Labtech, Offenburg, Germany). The ferric reducing/antioxidant power of each *D. longan* extract was expressed in the form of equivalent concentration (EC_1_), representing the ferric-TPTZ reduction capacity, which is equivalent to 1 mg of the *D. longan* extract. The EC_1_ values were calculated following Eq. (7);

7 $${\text{X}} = {{\left( {{\text{Y}} - 0.0{287}} \right)} \mathord{\left/ {\vphantom {{\left( {{\text{Y}} - 0.0{287}} \right)} {0.0{14}0{5}\left( {{\text{R}}^{{2}} = 0.{9926}} \right)}}} \right. \kern-\nulldelimiterspace} {0.0{14}0{5}\left( {{\text{R}}^{{2}} = 0.{9926}} \right)}},$$

where X is EC_1_ value and Y is an absorbance of each sample tested in the FRAP assay. l-Ascorbic acid was used as a positive control. The experiments were performed in triplicate.

#### Anti-inflammatory activity determination of dried *D. longan* and black* D. longan* extracts

Murine monocyte-macrophage (RAW 264.7) cells (American Type Culture Collection, ATCCTIB-71) treated with LPS were used to investigate the effect of *D. longan* extracts and their chemical compositions on the pro-inflammatory cytokine secretion (IL-6 and TNF-$\alpha$). Cells were cultured according to a method previously described with some modifications^47,48^. Briefly, RAW 264.7 cells with a density of 2 × 10^6^ cells per well in 750 μL of DMEM supplemented with GlutaMAX™-I, inactivated FBS (10%), penicillin (100 U/mL), streptomycin (100 μg/mL), and amphotericin B (0.25 μg/mL) were seeded in wells of 24 well-plates and incubated in a CO_2_ incubator (37 °C, 5% CO_2_ in air, 90% humidity) for 24 h. Thereafter, 1 µL of the *D. longan* extracts or dexamethasone (100 µg/mL) was added and further incubated in a CO_2_ incubator (37 °C, 5% CO_2_ in air, 90% humidity). Three replicates per sample were performed. After 24 h of the extract treatment, 250 μL of LPS in DMEM (4 µg/mL) was treated in each well and incubated in a CO_2_ incubator (37 °C, 5% CO_2_ in air, 90% humidity) for another 24. After that, the treated cells along with the supernatant were divided into two parts. The medium (500 μL) was collected for cytokine dosages, while the attached cells were subjected to the viability assay using MTT dye. The collected medium was then centrifuged for 10 min at 13,500×*g*, and the supernatant was investigated for cytokine secretion using an enzyme-linked immunosorbent assay (ELISA) following the manufacturer’s protocol (R&D Systems). The remain medium, which was left over in the wells, was investigated for cell viability using a 3-(4,5-dimethylthiazol-2-yl)-2,5-diphenyltetrazolium bromide (MTT) assay. To reduce variation due to cell density differences, secretion of IL-6 and TNF-α from RAW 264.7 cells was normalized to MTT levels^48^. RAW 264.7 cells without lipopolysaccharide treatment served as a negative control, while 100% of cytokine secretion was from the positive control of RAW 264.7 cells treated with LPS. The inhibitory activities of each sample were calculated following Eq. (8);

8 $$\% \;{\text{Cytokine}}\;{\text{inhibition}} = 100{-}{\text{A}}$$

where A is the cytokine secretion. Dexamethasone served as a positive control for both IL-6 and TNF-α secretory inhibition. The experiments were performed in triplicate.

#### Anti-hyaluronidase activity determination of dried *D. longan* and black* D. longan* extracts

The hyaluronidase inhibitory activity of *D. longan* extracts, gallic acid, corilagin, and ellagic acid was evaluated by measuring a product from the cleavage of sodium hyaluronate by hyaluronidase^49^. Prior to the experiment, the enzyme activity of hyaluronidase was determined. More than 90% enzyme activity was used in the anti-hyaluronidase activity determination. Firstly, 20 μL of the ethanolic solution of *D. longan* extracts (1 mg/mL) was mixed with hyaluronidase (15 unit/mL). After incubation at 37 °C for 10 min, hyaluronic acid solution (0.03 % w/v) in phosphate buffer pH 5.35 was added and further incubated for 45 min. Immediately after the addition of acid bovine serum albumin, the extracts were measured for an absorbance at 600 nm using a multimode detector (SPECTROstar Nano, BMG Labtech, Offenburg, Germany). The hyaluronidase inhibitory activity was calculated according to Eq. (9);

9 $$\% \;{\text{Inhibition}} = \left[ {\left. {1 - {\text{X}}/{\text{Y}}} \right)} \right] \times 100,$$

where X is the absorbance of the mixtures with sample; Y is the absorbance of the mixtures without sample. Oleanolic acid was used as a positive control. The experiments were performed in triplicate.

### Statistical analysis

All the values are given as means ± standard deviations and were analyzed. The statistical analysis used was one-way analysis of variance (ANOVA) followed by Tukey's post-hoc tests using the SPSS software (SPSS Statistics 21.0, IBM Corporations, New York, NY, USA). A value of p < 0.05 was accepted as significant.
